# An Autoantigen Profile of Human A549 Lung Cells Reveals Viral and Host Etiologic Molecular Attributes of Autoimmunity in COVID-19

**DOI:** 10.1101/2021.02.21.432171

**Published:** 2021-02-22

**Authors:** Julia Y. Wang, Wei Zhang, Michael W. Roehrl, Victor B. Roehrl, Michael H. Roehrl

**Affiliations:** 1 Curandis, New York, USA; 2 Department of Gastroenterology, Affiliated Hospital of Guizhou Medical University, Guizhou, China; 3 Department of Pathology, Memorial Sloan Kettering Cancer Center, New York, USA; 4 Human Oncology and Pathogenesis Program, Memorial Sloan Kettering Cancer Center, New York, USA

**Keywords:** COVID-19, autoimmunity, autoantigens, lung

## Abstract

We aim to establish a comprehensive COVID-19 autoantigen atlas in order to understand autoimmune diseases caused by SARS-CoV-2 infection. Based on the unique affinity between dermatan sulfate and autoantigens, we identified 348 proteins from human lung A549 cells, of which 198 are known targets of autoantibodies. Comparison with current COVID data identified 291 proteins that are altered at protein or transcript level in SARS-CoV-2 infection, with 191 being known autoantigens. These known and putative autoantigens are significantly associated with viral replication and trafficking processes, including gene expression, ribonucleoprotein biogenesis, mRNA metabolism, translation, vesicle and vesicle-mediated transport, and apoptosis. They are also associated with cytoskeleton, platelet degranulation, IL-12 signaling, and smooth muscle contraction. Host proteins that interact with and that are perturbed by viral proteins are a major source of autoantigens. Orf3 induces the largest number of protein alterations, Orf9 affects the mitochondrial ribosome, and they and E, M, N, and Nsp proteins affect protein localization to membrane, immune responses, and apoptosis. Phosphorylation and ubiquitination alterations by viral infection define major molecular changes in autoantigen origination. This study provides a large list of autoantigens as well as new targets for future investigation, e.g., UBA1, UCHL1, USP7, CDK11A, PRKDC, PLD3, PSAT1, RAB1A, SLC2A1, platelet activating factor acetylhydrolase, and mitochondrial ribosomal proteins. This study illustrates how viral infection can modify host cellular proteins extensively, yield diverse autoantigens, and trigger a myriad of autoimmune sequelae.

## Introduction

To gain better understanding of the transient and chronic autoimmune symptoms caused by SARS-CoV-2 infection, we have embarked on an endeavor to establish a comprehensive autoantigenome for COVID-19. In a previous study, we identified a repertoire of autoantigens (autoAgs) from human fetal lung fibroblast HFL1 cells that are strongly tied to neurological and diverse autoimmune symptoms of COVID-19 (1). In this study, we aim to identify additional autoAgs from human lung epithelium-like A549 cells, an adenocarcinoma cell line that is frequently used as a model host in SARS-CoV-2 infection studies.

AutoAgs were identified based on the unique affinity between autoAgs and the glycosaminoglycan dermatan sulfate (DS) that we have discovered (2, 3). AutoAgs and DS form affinity complexes that can engage strong dual BCR signaling in autoreactive B1 cells to induce autoantibody production (4). Hence, any self-molecule capable of forming affinity complexes with DS has a high propensity to become autoantigenic. This unifying mechanism of autoantigenicity explains how seemingly unrelated self-molecules can all induce autoimmune B cell responses via a similar immunological signaling event. Based on DS-autoAg affinity, we have cataloged several hundred autoAgs from various cells and tissues (1, 5–7).

COVID-19 is accompanied by a wide range of autoimmune symptoms, including multisystem inflammatory syndrome in children, immune thrombocytopenic purpura, antiphospholipid syndrome, autoimmune cytopenia, immune-mediated neurological syndromes, Guillain-Barré syndrome, connective tissue disease-associated interstitial lung disease, autoimmune hemolytic anemia, autoimmune encephalitis, systemic lupus erythematosus, optic neuritis and myelitis, and acquired hemophilia (8–15). Numerous autoantibodies have been identified in COVID patients, including the classical ANA (antinuclear antibody) and ENA (extractable nuclear antigen) that are hallmarks of systemic autoimmune diseases, as well as others such as anti-neutrophil cytoplasmic antibody, lupus anticoagulant, antiphospholipid, anti-IFN, anti-myelin oligodendrocyte glycoprotein, and anti-heparin-PF4 complex antibodies (8–15).

SARS-CoV-2, or viruses in general, are opportunistic intracellular pathogens that rely on the host for replication and survival. They hijack the host transcription and translation machinery for their replication, they compromise the host immune defense to evade destruction, and they modulate the host cell cycle and apoptosis for symbiosis. These viral processes are accomplished through extensive modification of host cellular components, which also results in changes in self-molecules and the emergence of autoAgs. In our previous studies, we reported that self-molecules derived from apoptotic cells display strong affinity to DS, becoming a major source of autoAgs (2, 3). In this study, we report several important molecular mechanisms in SARS-CoV-2 infection that change host self-molecules to autoAgs, including direct interaction with viral components, perturbation by viral protein expression, and post-translational protein modification by ubiquitination and phosphorylation from viral infection.

## Results and Discussion

### A putative A549 autoantigenome identified by DS-affinity

By DS-affinity fractionation and mass spectrometry sequencing, we identified a global putative autoantigenome of 348 proteins from A549 cellular protein extracts, with 214 protein having strong affinity and 134 having intermediate affinity (Table 1). To find out whether these DS-affinity proteins are known autoAgs, we conducted an extensive literature search and confirmed that 198 (56.0%) proteins are known humoral autoAgs, with their specific autoantibodies reported in a wide spectrum of autoimmune diseases and cancers (see autoAg confirmatory references in Table 1). The remaining 150 proteins may be yet-to-be discovered putative autoAgs and await further investigation. For example, many ribosomal proteins are known autoAgs, but the 24 mitochondrial ribosomal proteins we identified have not yet been reported as autoAgs; given their structural similarity to ribosomal protein autoAgs, it is highly likely that mitochondrial proteins are a group of undiscovered autoAgs.

**Fig. 1.**
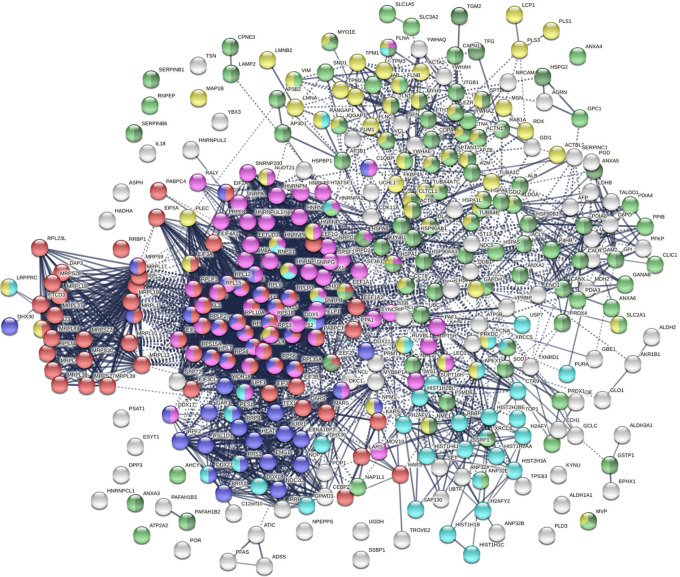
The autoantigenome from A549 cells identified by DS affinity. Marked proteins are associated with translation (69 proteins, red), mRNA metabolic processing (69 proteins, pink), ribosome biogenesis (43 proteins, blue), vesicle (87 proteins, green) and vesicle-mediated transport (72 proteins, dark green), chromosomes (40 proteins, aqua), and cytoskeleton (65 proteins, gold).

The 348 DS-affinity proteins are highly connected (Fig. 1). They exhibit 6,271 interactions, whereas a random set of 348 proteins is expected to have 2,536 interactions, as revealed by protein-protein interaction STRING analysis (16). The tight connections suggest that these known and putative autoAgs are originating from common biological pathways or processes. Our analysis shows that they are indeed predominantly associated with translation, mRNA metabolic process, ribonucleoprotein complex biogenesis, vesicle and vesicle-mediated transport, chromosome, and cytoskeleton (Fig. 1).

### COVID-altered proteins among the A549 autoantigenome

To find out whether the known and putative autoAgs identified by DS-affinity may play a role in SARS-CoV-2 infection, we compared our A549 autoantigenome with currently available COVID data compiled in the Coronascape database (17–37). Of our 348 autoantigenome proteins from A549 cells, 291 (83.6%) have been found to be COVID-altered, i.e., up- and/or down-regulated at protein and/or mRNA level in SARS-CoV-2 infected cells or patient tissues (Table 1). Because the COVID data have been generated from various research labs using different techniques and sources of cells or tissues, 190 proteins are found to be up in some studies but down in others. In total, 231 proteins are found up-regulated, and 252 are found down-regulated in SARS-CoV-2 infection. Based on reported autoantibodies, 191 (65.6%) COVID-altered proteins are confirmed autoAgs (Table 1).

Based on gene ontology (GO) cellular component analysis, proteins of the A549 DS-affinity autoantigenome that are also altered in COVID infection can be located to membrane-bound organelles (247 proteins), nucleus (177 proteins), ribonucleoprotein complex (95 proteins), mitochondrion (46 proteins), endoplasmic reticulum (45 proteins), secretory granules (41 proteins), melanosome (27 proteins), myelin sheath (28 proteins), and axon (16 proteins).

**Fig. 2.**
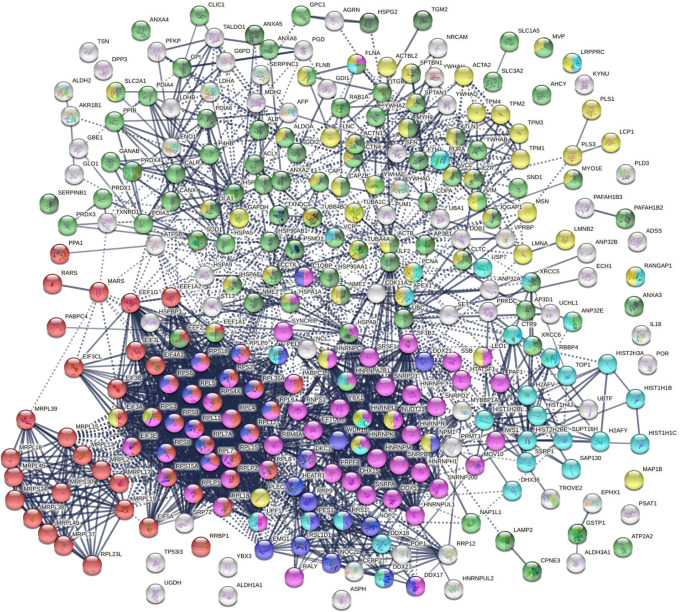
COVID-altered proteins shared with the A549 autoantigenome. Marked proteins are associated with translation (53 proteins, red), mRNA metabolic process (63 proteins, pink), vesicle (79 proteins, green) and vesicle-mediated transport (64 proteins, dark green), cytoskeleton (58 proteins, gold), chromosomes (35 proteins, aqua), and ribosome biogenesis (29 proteins, blue).

**Fig. 3.**
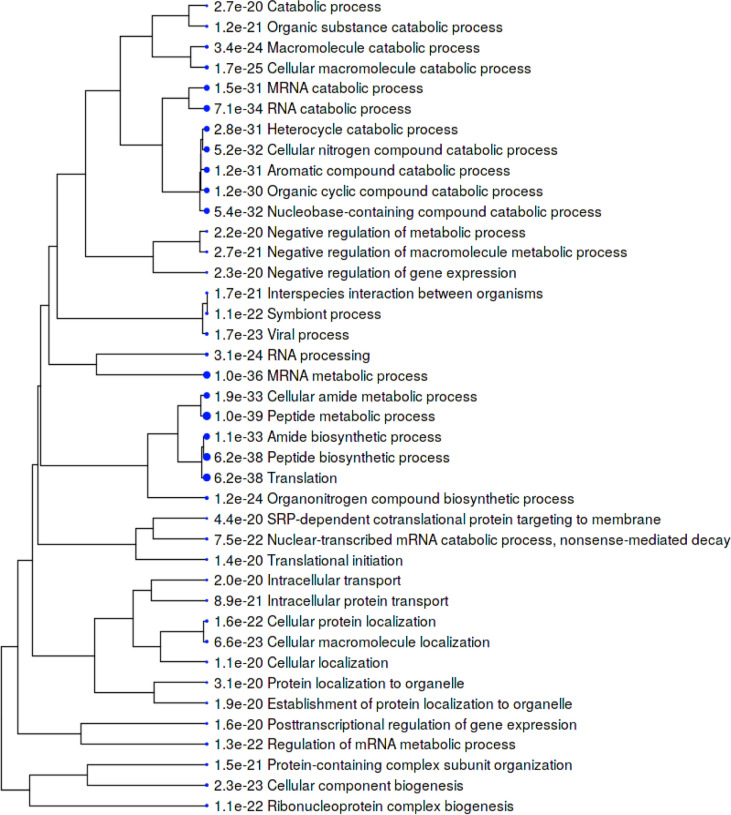
Top 40 enriched GO biological processes among COVID-altered proteins shared with the A549 autoantigenome. Bigger dots indicate more significant P-values.

Within the total A549 autoantigenome, the 291 COVID-altered proteins form a tightly interacting network (Fig. 2). At high STRING protein-protein interaction confidence level, these proteins exhibit 2,249 interactions, whereas 953 interactions would be expected of a random collection of proteins of the same size. By GO biological process analysis, the COVID-altered proteins are significantly enriched in various biological processes, including translation, peptide biosynthetic process, RNA catabolic process, nucleobase-containing compound catabolic process, SRP-dependent cotranslational protein targeting to membrane, protein localization to organelle, and symbiont process. Among these processes associated with COVID-altered proteins, the hierarchical cluster tree root points to ribonucleoprotein complex biogenesis (Fig. 3).

**Fig. 4.**
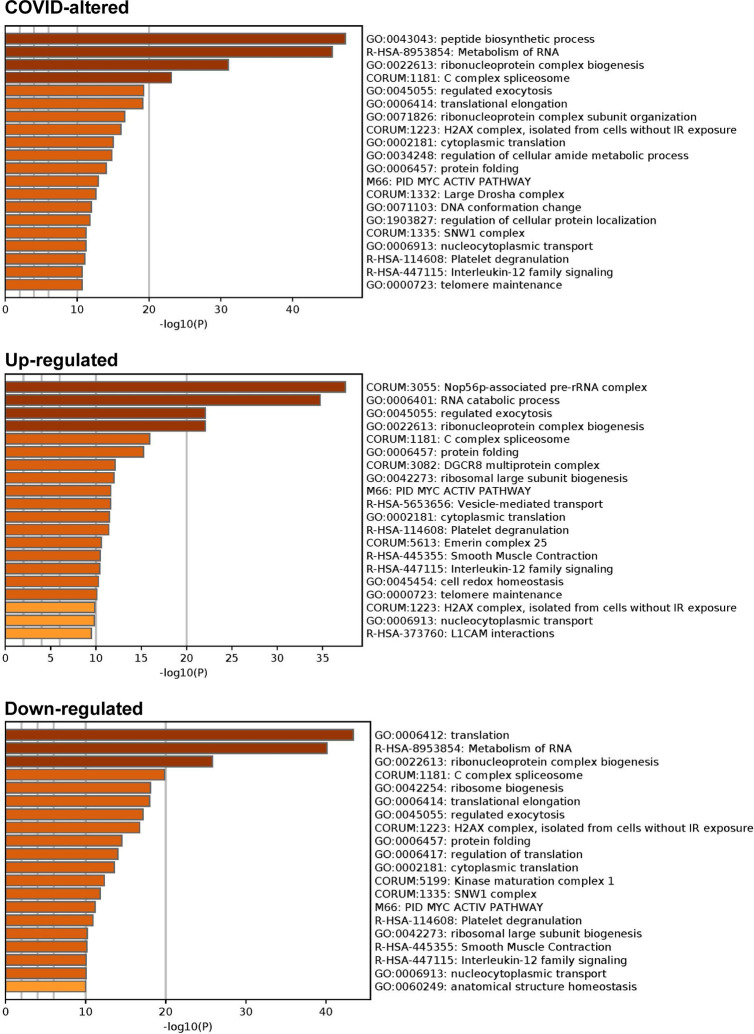
Top 20 enriched pathways and processes among COVID-altered autoAgs. Top: 298 COVID-altered autoAgs. Middle: 231 up-regulated autoAgs in COVID. Bottom: 252 down-regulated autoAgs in COVID.

Combined pathway and process enrichment analyses also show that the COVID-altered DS-affinity proteins are most significantly related to peptide biosynthetic process, metabolism of RNA, and ribonucleoprotein complex biogenesis (Fig. 4). The up-regulated autoAgs are more related to Nop56p-associated pre-rRNA complex, RNA catabolic process, and vesicle-mediated transport, whereas the down-regulated autoAgs are more related to translation and ribosome biogenesis. COVID-altered autoAgs are also significantly associated with regulated exocytosis, platelet degranulation, smooth muscle contraction, and IL-12 signaling.

The molecular functions of the COVID-altered DS-affinity autoAgs include RNA binding (76 proteins), hydrolase activity (58 proteins), purine ribonucleotide triphosphate binding (55 proteins), pyrophosphatase activity (35 proteins), nucleoside-triphosphatase activity (34 proteins), oxidoreductase activity (26 proteins), ATPase activity (25 proteins), ubiquitin protein ligase binding (21 proteins), mRNA 3’-UTR binding (11 proteins) and 5’-UTR binding (3 proteins), and helicase activity (13 proteins).

### AutoAgs that interact with SARS-CoV-2 proteins

To study the origins of COVID-induced autoAg alterations, we examined DS-affinity proteins that are involved in the interactomes of SARS-CoV-2 viral proteins (19, 30, 34) and found 38 DS-affinity proteins that interact directly with different viral proteins, with 25 of them being known autoAgs, e.g., ATP5B, CANX, DDX21, EEF1A2, PDIA3, and SLC2A1 (Table 1). Orf3 protein is the most striking, as its interactome includes 14 DS-affinity proteins and 9 known autoAgs. N protein interacts with 9 DS-affinity proteins and 6 known autoAgs, including 2 helicases (DDS21 and MOV10), 2 poly(A)-binding proteins (PABPC1 and PABPC4). Nsp4 interact with 6 DS-affinity proteins and 5 known autoAgs. Nsp4 interacts with IDE (insulin degrading enzyme), which is not a known autoAg.

A few host DS-affinity proteins interact with more than one SARS-CoV-2 protein. RAB1A (involved in intracellular membrane trafficking) interacts with 3 viral proteins (Orf3, Orf7b, and Nsp7), and PLD3 (phospholipase) also interacts with 3 viral proteins (Nsp2, Orf7b, and Orf8), but neither RAB1A nor PLD3 has been discovered as autoAgs. HSPA5 (GRP78/BiP) interacts with Nsp2 and Nsp4, HSPA1A interacts with N and Orf9b, and both heat shock proteins are known autoAg. PRKDC, a known autoAg, interacts with M and Nsp. Interestingly, of the ezrin-radixin-moesin protein family that connects the actin cytoskeleton to the plasma membrane, RDX is found to interact with Orf3 and Nsp13, MSN interacts with Orf3 and Nsp6, and EZR is COVID-altered. Furthermore, RDX, MSN, and EZR are all known autoAgs (Table 1).

### AutoAgs from perturbation by viral protein expression

**Fig. 5.**
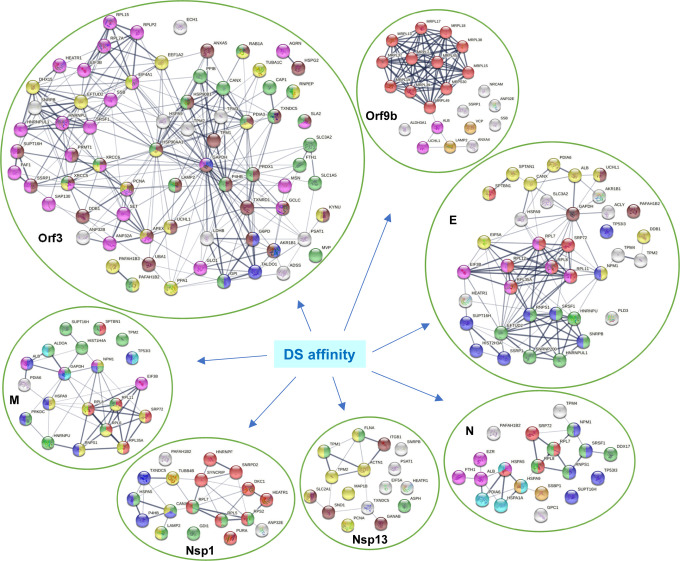
DS-affinity proteins that interact with SARS-CoV-2 viral proteins or are perturbed in A549 cells expressing individual viral proteins. **Orf3:** regulation of gene expression (pink), cytoplasmic vesicle (green), monosaccharide biosynthetic process (blue), response to stress (brown), and hydrolase activity (gold). **Orf9b:** mitochondrial translation (red), mitochondrion localization (pink), autophagosome maturation (gold). **E:** establishment of protein localization to membrane (red), translation initiation (pink), mRNA splicing (green), regulation of macroautophagy (brown), post-translational protein modification (gold), RNA polymerase II transcription (blue). **M:** establishment of protein localization to membrane (red), intracellular protein transport (gold), organelle organization (green), nicotinamide nucleotide metabolic process (aqua), regulation of apoptotic process (blue), and symbiont process (pink). **N:** maintenance of location in cell (pink), protein localization to ER (red), protein folding (aqua), mitochondrial nucleoid (amber), RNA polymerase II transcription (blue), and RNA-binding (green). **Nsp1:** protein localization (green), gene expression (red), protein processing in ER (blue), and phagosome (gold). **Nsp13:** cytoskeleton (gold), regulation of muscle contraction (green), and melanosome (brown).

To find out how individual SARS-CoV-2 proteins affect the host, Stukalov et al. conducted extensive proteomic analysis of A549 cells transduced to express individual SARS-CoV-2 proteins (34). By comparing with their data, we identified 167 DS-affinity proteins that are perturbated by viral protein expression in A549 cells. Among all SARS-CoV-2 proteins, Orf3 expression produced the largest number of potential autoAgs, with 26 up- and 36 down-regulated being DS-affinity proteins (Fig. 5, Supplemental Table 1). Other viral protein expressions affected various numbers of DS-affinity proteins, including E (20 up and 18 down), Orf9b (6 up and 16 down), M (10 up and 11 down), N (10 up and 8 down), Nsp13 (5 up and 11 down), and Nsp12 (3 up and 12 down). Interestingly, S expression yielded only 3 up- and 2 down-regulated DS-affinity proteins, suggesting that it may not have much intracellular activity.

In total, Orf3 affected 71 DS-affinity proteins identified from A549 cells, which includes those directly interacting with Orf3 and those perturbed by Orf3 protein expression in A549 cells. The large number of Orf3-affected host proteins implicates important roles of Orf3 in SARS-CoV-2 infection. Network analysis reveals these proteins to be mostly associated with gene expression regulation, cytoplasmic vesicles, apoptosis, response to stress, monosaccharide biosynthesis, or hydrolase activity (Fig. 5). Several of these are classical nuclear autoAgs, e.g., PNCA, SSB (Lupus La), XRCC5 (Lupus Ku80, thyroid-lupus autoAg), XRCC6 (Ku70), and SNRPB (SmB/B’). A few are unknown autoAgs but with important relevance to COVID, e.g., PAFAH1B2 and PAFAH1B3 (the alpha catalytic subunits of the cytosolic type I platelet-activating factor (PAF) acetylhydrolase). PAF is produced by a variety of cells involved in host defense, and PAF signaling can trigger inflammatory and thrombotic cascades. The modulation of PAF by SARS-CoV-2 Orf3 may partially explain the frequently occurring thrombotic complications and coagulopathy in COVID-19 patients. PAF also induces apoptosis in a PAF receptor independent pathway that can be inhibited by PAFAH1B2 and PAFAH1B3 (38).

SARS-CoV-2 E protein affects a number of ribonucleoproteins that are related to translation initiation and mRNA splicing, e.g., hnRNP (U and UL1) and ribosomal protein (L7, L8 L11, L12, L35A). E-affected proteins are associated with establishment of protein localization to membrane, regulation of autophagy, and post-translational protein modification (Fig. 5). SARS-CoV-2 M, Nsp1, and N proteins also affect various ribonucleoproteins, whereas Nsp13 appears to affect proteins associated with the cytoskeleton. Overall, the majority of DS-affinity proteins found affected by individual SARS-CoV-2 proteins are known autoAgs (Fig. 5 and Table 1), which indicates that host proteins perturbed by viral components are an important source of autoAgs.

### Mitochondrial perturbation by SARS-CoV-2 Orf9b

By DS-affinity fractionation, we identified 22 mitochondrial ribosomal proteins from A549 cells with strong DS-affinity, including mitochondrial 39S ribosomal proteins (L1, L2, L13, L15, L17, L18, L19, L23, L37, L38, L39, L45, L49) and 28S ribosomal proteins (S9, S22, S23, S27, S28, S29, S30, S34, S39) (Table 1). Remarkably, 14 mitochondrial ribosomal proteins are found down-regulated in SARS-CoV-2 infection, with 2 (L15 and L37) reported both down- and up-regulated. Moreover, MRPS27 is found in the interactome of SAS-Cov-2 Nsp8. Most strikingly, Orf9b-expression in A549 cells caused down-regulation of 16 proteins that we identified by DS-affinity, namely, ALB, ANXA4, LAMP2, MRPL2, MRPL13, MRPL15, MRPL17, MRPL18, MRPL19, MRPS30, MRPL37, MRPL38, MRPL39, MRPL45, MRPL49, and UCHL1 (Fig. 5). Eleven of the Orf9-affected proteins are mitochondrial ribosomal proteins, which may affect the mitochondrial translation machinery. Orf9-affected proteins may also be involved in mitochondrion localization, autophagosome maturation, or other processes. Overall, these findings suggest that SARS-CoV-2 infection may affect mitochondria primarily through Orf9b.

Orf9b of SARS-CoV has been shown to localize to mitochondria, trigger ubiquitination and proteasomal degradation of dynamin-like protein 1, limit host cell interferon signaling by targeting mitochondrial associated adaptor molecule MAVS signalosome, and manipulate the mitochondrial function to help evade host innate immunity (39). Orf9b of SARS-CoV-2 has been reported to suppress the type I interferon response by targeting TOM70 (40). In COVID-19 pneumonia patients, monocytes show altered bioenergetics and mitochondrial dysfunction with depolarized and abnormal ultrastructure (41).

Currently, little is known about the involvement of mitochondrial ribosomes or mitochondrial translation in SARS-CoV-2 infection. Expression of mitochondrial ribosomal proteins associated with protein synthesis has been found to be the most striking transcriptional difference among dengue virus-infected children, as revealed by a genome-wide microarray analysis of whole blood RNA from 34 infected children collected on days 3–6 of illness (42). In human cytomegalovirus infection, proteins involved in biogenesis of the mitochondrial ribosome changed early during the viral replication cycle (43). Mitochondria are vital to cell survival and apoptosis as they produce the majority of adenosine triphosphate (ATP) that provide chemical energy to cells. Especially for cells such as muscles that require much ATP, mitochondrial dysfunction will certainly lead to problems such as muscle weakness and fatigue. The roles of mitochondrial ribosomal proteins play in COVID and long-term sequelae merit further investigation.

### AutoAgs related to ubiquitination alteration in SARS-CoV-2 infection

**Fig. 6.**
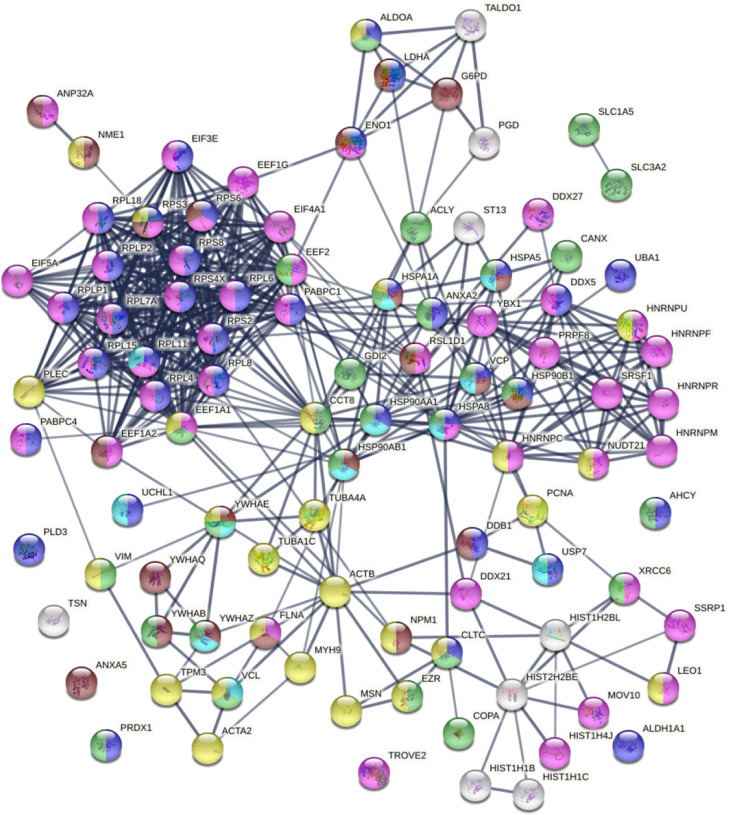
Known and putative autoAgs derived from ubiquitination alteration in SARS-CoV-2 infected AF549 cells. Marked proteins are associated with catabolic process (37 proteins, blue), ubiquitin protein ligase binding (12 proteins, aqua), gene expression (46 proteins, pink), regulation of apoptotic process (22 proteins, brown), cytoplasmic vesicles (28 proteins, green), and cytoskeleton (26 proteins, gold).

Ubiquitination provides a universal signal for protein degradation. By comparing our data with the ubiquitinome of SARS-CoV-2 infected cells, we identified 102 DS-affinity proteins that are altered by ubiquitination during viral infection (Supplemental Table 1). These ubiquitination-altered proteins are significantly associated with gene expression, catabolic process, regulation of apoptotic process, cytoplasmic vesicles, and cytoskeleton (Fig. 6). They include 15 ribosomal proteins, 8 heat shock proteins, 5 hnRNP proteins, 5 histones, 4 translation elongation factors, and 3 translation initiation factors, and a majority of them are known autoAgs (Table 1).

Three ubiquitination/de-ubiquitination enzymes (UBA1, UCHL1, and USP7) are COVID-altered and possess DS-affinity, with UBA1 and UCHL1 being known autoAgs. UBA1 catalyzes the first step in ubiquitin conjugation to mark proteins for degradation through the ubiquitin-proteasome system. USP7 is a hydrolase that deubiquitinates target proteins. UCHL1 is a thiol protease that recognizes and hydrolyzes a peptide bond at the C-terminal glycine of ubiquitin, and is involved in the processing of ubiquitin precursors and of ubiquitinated proteins. UBA1 is found down-regulated by Orf3 expression. UCHL1 is found in the Orf3 interactome, up-regulated by SARS-CoV-2 E protein expression, and down-regulated by Nsp12, Nsp8, Orf8, and Orf9b (Supplemental Table 1).

Ten COVID-altered DS-affinity proteins were identified with ubiquitin protein ligase-binding activity, including HSPA1A, HSPA5, HSPA8, HSP90AA1, HSP90AB1, RPL11, VCP, VCL, YWHAE, and YWHAZ. HSPA1A interacts with SARS-CoV-2 Orf9b and N proteins, HSPA5 (GRP78/BiP) interacts with Nsp2 and Nsp4, and HSPA8 interacts with Nsp2. Except for RPL11, all are known autoAgs (Table 1).

In addition, SARS-CoV-2 and other coronaviruses encode for papain-like proteases (PLP), an important multifunctional enzyme with de-ubiquitination, de-ISGlation, and interferon antagonism activities (44). PLPs, along with other proteases, are responsible for processing replicase proteins that are required from viral replication. PLP of SARS-CoV-2 is able to reverse host ubiquitination and remove interferon-stimulated gene product 15 (ISG15), and its substrate activity mirrors closely that of PLP of MERS (45). Ubiquitin modifications can regulate innate immune response and apoptosis, and ISG15 is a ubiquitin-like modifier typically expressed during host cell immune response. Overall, various components of SARS-CoV-2 appear to be able to alter uniquitination of host proteins. The large pool of ubiquitin-altered proteins in SARS-CoV-2 infection indicates that ubiquitin modification, such as differential abundance and dynamic ubiquitination pattern change, may be a major origin of autoAgs.

### AutoAgs related to phosphorylation alteration in SARS-CoV-2 infection

**Fig. 7.**
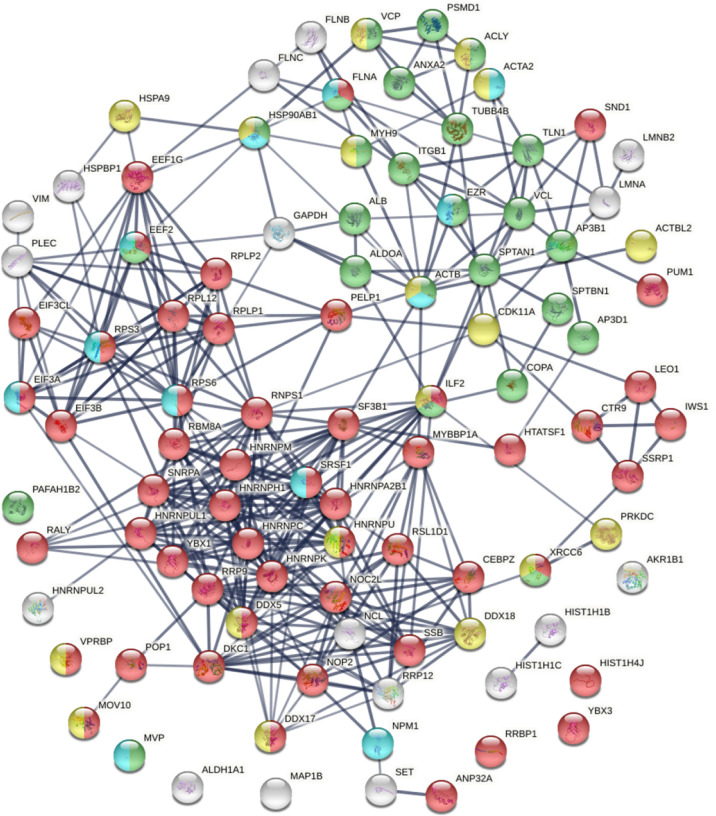
Known and putative autoAgs derived from phosphorylation alteration in SARS-CoV-2 infected cells. Marked proteins are associated with gene expression (52 proteins, red), vesicle-mediated transport (25 proteins, green), ATP binding (18 proteins, gold), and kinase binding (12 proteins, aqua).

Comparing our data with currently available phosphoproteome data of COVID-19 (26, 34), 97 phosphoproteins are found with both DS-affinity and COVID-induced alteration, with a majority (52/97) related to gene expression (Fig. 7). Notably, they include 8 heterogeneous nuclear ribonucleoproteins affected by phosphorylation (HNRNPA2B1, HNRNPC, HNRNPH1, HNRNPK, HNRNPM, HNRNPU, HNRNPUL1, HNRNPUL2), with 4 being known autoAgs (Table 1). HNRNPs are involved in many cellular processes, including gene transcription, cell cycle, DNA damage control, post-transcriptional modification of newly synthesized pre-mRNA, and virus replication. For example, HNRNPA2B1 displays RNA-binding affinity to murine hepatitis coronavirus via binding the viral RNA at the 3’ end and modulate viral RNA synthesis (46).

There are 25 phosphorylation-altered proteins that are related to vesicle-mediated transport, most of which are known autoAgs, including ACLY, ACTA2, ACTB, ALB, ALDO, ANXA2, FLNA, COPA, SPTAN1, SPTBN1, TLN1, TUBB4, and VCL (Fig. 7 and Table 1). The coatomer, to which COPA (coatomer subunit alpha) belongs, is a cytosolic protein complex that associates with Golgi non-clathrin-coated vesicles and is required for budding from the Golgi membrane. COPA is associated with autoimmune interstitial lung, joint, and kidney disease (47).

There are 18 phosphorylation-altered potential autoAgs with ATP binding activity, and 12 with kinase binding activity (Fig. 7). In particular, PRKDC (DNA-dependent protein kinase catalytic subunit) is identified with strong DS-affinity and is a known autoAg. It is a serine/threonine-protein kinase that acts as a molecular sensor for DNA damage, with involvement in numerous biological processes such as DNA damage and repair, immunity, innate immunity, ribosome biogenesis, and apoptosis. PRDKC is found in the interactomes of M and Nsp4 proteins of SARS-CoV-2 and up-regulated by expression of Nsp10, Nsp9, Orf7a, or Orf7b protein in A549 cells (19, 34). PRKDC is also found up-regulated at 0 h and 4 h in SARS-CoV-2 infected Vero E6 cells (26) and up-regulated at 24 h in SARS-CoV-2 infected Caco-2 cells (20). These findings suggest that phosphorylation by PRKDC plays extensive and important roles in COVID.

Proteins phosphorylated during apoptosis are common targets of autoantibodies. For example, the U1–70 snRNP autoAg undergoes specific changes in the phosphorylation/dephosphorylation balance and cellular localization during apoptosis (48), and phosphorylated U1-snRNP complex induced by apoptosis is recognized by autoantibodies in patients with systemic lupus erythematosus (49). A high degree of phosphorylation of SSB (lupus La autoAg) substantially diminished its poly(U) binding capacity, but its binding to human autoantibodies increased 2-fold with increased phosphorylation (50). On the other hand, SSB autoAg has also been reported to be dephosphorylated and cleaved during early apoptosis (51). During apoptosis, ribosomal protein P1 and P2 autoAgs are completely dephosphorylated while P0 autoAg is partially dephosphorylated (52). Therefore, alterations in phosphorylation, either hyper- or hypophosphorylation, may lead to changes in self-molecules and render them autoantigenic.

## Conclusion

In our quest for a comprehensive autoantigen atlas for COVID-19, we report an autoantigen profile of 191 confirmed autoAgs and 150 putative autoAgs in SARS-CoV-2 infection. These proteins are initially identified from human lung epithelial A549 cells using a unique DS-affinity autoAg enrichment strategy, and then compared with currently available COVID-omics data. Our study reveals that cellular processes and components integral to viral infection are major origins of autoAgs, including gene expression, ribonucleoprotein biogenesis, translation and mitochondrial translation, vesicle and vesicle-mediated transport, and cytoskeleton. Ubiquitination and phosphorylation are particular post-translational modifications that cause changes in self-molecules and render them autoantigenic. Impaired clearance of apoptotic and dead cell material is considered a major pathogenic attribute to autoimmune disease. We have previously shown that DS possesses unique affinity to apoptotic cells and their released autoAgs, and our current study further demonstrates that ubiquitination and phosphorylation associated with apoptosis are possibly major sources of molecular alterations in self-molecule to autoantigen transformation. Overall, our study demonstrates that SARS-CoV-2 causes extensive alterations of host cellular proteins and produces a large number of potential autoAgs, indicating that there may be an intimate relationship between COVID infection and autoimmunity.

## Materials and Methods

### A549 cell culture

The A549 cell line was obtained from the ATCC (Manassas, VA, USA) and cultured in complete F-12K medium at 37 °C in 75 cm^2^ flasks to 80% confluency. The growth medium was supplemented with 10% fetal bovine serum and a penicillin-streptomycin-glutamine mixture (Thermo Fisher).

### Protein extraction

About 100 million A549 cells were suspended in 10 ml of 50 mM phosphate buffer (pH 7.4) containing the Roche Complete Mini protease inhibitor cocktail. Cells were homogenized on ice with a microprobe sonicator until the turbid mixture turned nearly clear with no visible cells left. The homogenate was centrifuged at 10,000 g at 4 °C for 20 min, and the total protein extract in the supernatant was collected. Protein concentration was measured by absorbance at 280 nm using a NanoDrop UV-Vis spectrometer (ThermoFisher).

### DS-Sepharose resin preparation

The DS-affinity resins were prepared as previously described (3, 5). In brief, 2 ml of EAH Sepharose 4B resins (GE Healthcare Life Sciences) were washed with distilled water three times and mixed with 10 mg of DS (Sigma-Aldrich) in 1 ml of 0.1 M MES buffer, pH 5.0. About 20 mg of N-(3-dimethylaminopropyl)-N’-ethylcarbodiimide hydrochloride (Sigma-Aldrich) powder was added at the beginning of the reaction, and another 20 mg was added after 8 h of reaction. The reaction mixture was mixed by end-over-end rotation at 25 °C for 16 h. The coupled resins were washed with water three times and equilibrated with a low-pH buffer (0.1 M acetate, 0.5 M NaCl, pH 5.0) and a high-pH buffer (0.1 M Tris, 0.5 M NaCl, pH 8.0).

### DS-affinity fractionation

The total proteins extracted from A549 cells were fractionated in a DS-Sepharose column with a BioLogic Duo-Flow system (Bio-Rad). About 40 mg of proteins in 40 ml of 10 mM phosphate buffer (pH 7.4; buffer A) were loaded onto the column at a rate of 1 ml/min. Unbound and weakly proteins were washed off with 60 ml of buffer A and then 40 ml of 0.2 M NaCl in buffer A. The remaining bound proteins were eluted with 40 ml 0.5 M NaCl and then with 40 ml 1.0 M NaCl in buffer A. Fractions were desalted and concentrated to 0.5 ml with 5-kDa cut-off Vivaspin centrifugal filters (Sartorius). Fractionated proteins were separated by 1-D SDS-PAGE in 4–12% Bis-Tris gels, and the gel lanes were divided into two or three sections and subjected to sequencing.

### Mass spectrometry sequencing

Protein sequencing was performed at the Taplin Biological Mass Spectrometry Facility at Harvard Medical School. Proteins in gels were digested with sequencing-grade trypsin (Promega) at 4 °C for 45 min. Tryptic peptides were separated on a nano-scale C_18_ HPLC capillary column and analyzed in an LTQ linear ion-trap mass spectrometer (Thermo Fisher). Peptide sequences and protein identities were assigned by matching the measured fragmentation pattern with proteins or translated nucleotide databases using Sequest. All data were manually inspected. Only proteins with ≥2 peptide matches were considered positively identified.

### COVID data comparison

DS-affinity proteins were compared with currently available proteomic and transcriptomic data from SARS-CoV-2 infection compiled in the Coronascape database (as of 12/14/2020) (17–37). These data had been obtained with proteomics, phosphoproteomics, interactome, ubiquitome, and RNA-seq techniques. Up- and down-regulated proteins or genes were identified by comparing COVID-19 patients vs. healthy controls and cells infected vs. uninfected by SARS-CoV-2. Similarity searches were conducted between our data and the Coronascape database to identify DS-affinity proteins (or their corresponding genes) that are up- and/or down-regulated in the viral infection.

### Protein-protein interaction network analysis

Protein-protein interactions were analyzed by STRING (16). Interactions include both direct physical interaction and indirect functional associations, which are derived from genomic context predictions, high-throughput lab experiments, co-expression, automated text mining, and previous knowledge in databases. Each interaction is annotated with a confidence score from 0 to 1, with 1 being the highest, indicating the likelihood of an interaction to be true. Only interactions with high confidence (a minimum score of 0.7) are shown in the figures.

### Pathway and process enrichment analysis

Pathways and processes enrichment were analyzed with Metascape (17), which utilize various ontology sources such as KEGG Pathway, GO Biological Process, Reactome Gene Sets, Canonical Pathways, CORUM, TRRUST, and DiGenBase. All genes in the genome were used as the enrichment background. Terms with a p-value <0.01, a minimum count of 3, and an enrichment factor (ratio between the observed counts and the counts expected by chance) >1.5 were collected and grouped into clusters based on their membership similarities. The most statistically significant term within a cluster was chosen to represent the cluster. Hierarchical clustering trees were obtained with ShinyGo (53).

### Autoantigen confirmation literature text mining

Literature searches in Pubmed were performed for every DS-affinity protein identified in this study. Search keywords included the protein name, its gene symbol, alternative names and symbols, and the MeSH keyword “autoantibodies”. Only proteins with their specific autoantibodies reported in PubMed-listed journal articles were considered “confirmed” autoAgs in this study.

## Supplementary Material

Supplement 1

## Figures and Tables

**Table 1.** DS-affinity autoantigenome from human A549 cells

# Pep.	Gene	Protein	COVID		A549 infection		Interactome	DS-affinity		AutoAg ref.
			up	down	up	down		1.0 M	0.5 M	
2	A2M	Alpha-2-macroglobulin		D					+	(1)
2	ACLY	ATP-citrate synthase	U	D	U	D			+	(2)
9	ACTA2	Actin, aortic smooth muscle	U	D	U	D		+		(3)
8	ACTB	Actin, cytoplasmic 1	U	D	U	D		+		(4)
5	ACTBL2	Beta-actin-like protein 2	U	D	U	D		+		(4)
20	ACTN1	Alpha-actinin-1	U	D	U	D			+	(5)
20	ACTN4	Alpha-actinin-4	U	D	U				+	(3)
2	ADSS2	Adenylosuccinate synthetase isozyme 2	U		U				+	
3	AFP	Alpha-fetoprotein		D					+	(6)
2	AGRN	Agrin	U		U			+		(7)
6	AHCY	Adenosylhomocysteinase		D		D			+	
5	AKR1B1	Aldose reductase (diabetic complication)	U	D	U	D	Orf3		+	(8)
6	ALB	Putative uncharacterized protein ALB	U	D	U	D		+		(9)
23	ALDH1A1	Retinal dehydrogenase	U	D	U	D			+	(10)
5	ALDH2	Aldehyde dehydrogenase, mitochondrial	U	D					+	(11)
5	ALDH3A1	Aldehyde dehydrogenase, dimeric NADP-preferring, ALDH3	U	D	U	D			+	
9	ALDOA	Fructose-bisphosphate aldolase	U	D	U	D			+	(12)
6	ANP32A	Acidic leucine-rich nuclear phosphoprotein 32 family member A	U	D		D		+		
13	ANP32B	Acidic leucine-rich nuclear phosphoprotein 32 family member B, APRIL		D		D		+		(13)
3	ANP32C	Acidic leucine-rich nuclear phosphoprotein 32 family member C, PP32R1						+		
4	ANP32E	Acidic leucine-rich nuclear phosphoprotein 32 family member E	U	D	U	D		+		
4	ANXA2	Annexin A2	U	D	U				+	(14)
13	ANXA2P2	Annexin A2 pseudogene 2	U	D	U				+	(14)
10	ANXA3	Annexin A3	U	D	U				+	(15)
5	ANXA4	Annexin IV	U	D	U	D			+	(16)
15	ANXA5	Annexin A5	U	D	U	D	Orf3		+	(17)
8	ANXA6	Annexin VI	U	D					+	(18)
8	AP3B1	AP-3 complex subunit beta-1	U				E	+		
2	AP3B2	AP-3 complex subunit beta-2						+		(19)
8	AP3D1	AP-3 complex subunit delta-1	U	D				+		
4	APEX1	DNA-(apurinic or apyrimidinic site) endonuclease	U	D	U	D			+	(20)
4	ASPH	Aspartyl/asparaginyl beta-hydroxylase	U	D		D		+		
2	ATP2A2	ATP Sarcoplasmic/ER calcium transporting 2 (cardiac Ca2+ ATPase, heart failure)	U				Nsp4	+		(21)
13	ATP5B	ATP synthase subunit beta, mitochondrial, ATP5F1B	U	D			Nsp6	+		(22)
3	BRIX1	Ribosome biogenesis protein BRX1 homolog						+		
8	C1QBP	Complement C1q-binding protein, mitochondrial matrix protein p32		D		D		+		(23)
10	CALR	Calreticulin	U	D	U				+	(24)
7	CANX	Calnexin	U	D	U	D	Nsp4	+		(25)
2	CAP1	Cyclase associated actin cytoskeleton regulatory protein 1	U	D			Orf3		+	
2	CAPN1	Calpain-1 catalytic subunit							+	
2	CAPZB	F-actin-capping protein subunit beta		D					+	(26)
5	CCT8	T-complex protein 1 subunit theta	U	D	U	D			+	(27)
3	CDK11A	Cyclin-dependent kinase 11 A, CDC2L2	U					+		
3	CEBPZ	CCAAT/enhancer-binding protein zeta	U					+		
4	CLIC1	Chloride intracellular channel protein 1	U	D					+	(28)
4	CLTC	Clathrin heavy chain 1	U	D		D		+		(29)
2	CLTCL1	Clathrin heavy chain 2						+		
2	COPA	Coatomer subunit alpha	U	D	U	D		+		(30)
2	CPNE3	Copine-3	U	D					+	
2	CTR9	RNA polymerase-associated protein CTR9 homolog	U	D		D		+		
4	DCAF1	DDB1- and CUL4-associated factor 1, VPRBP	U	D	U			+		
13	DDB1	DNA damage-binding protein 1	U	D	U	D		+		(29)
3	DDX17	Probable ATP-dependent RNA helicase DDX17	U	D	U			+		
7	DDX18	ATP-dependent RNA helicase DDX18	U					+		
5	DDX21	Nucleolar RNA helicase 2 (RH II/Gu)	U	U			N	+		(31)
4	DDX27	Probable ATP-dependent RNA helicase DDX27	U		U			+		
3	DDX30	ATP-dependent RNA helicase DHX30, DDX30						+		
8	DDX48	Eukaryotic initiation factor 4A-III, EIF4A3						+		
4	DDX5	Probable ATP-dependent RNA helicase DDX5	U	D				+		
16	DDX9	ATP-dependent RNA helicase A, DHX9						+		(32)
12	DHX15	Putative pre-mRNA-splicing factor ATP-dependent RNA helicase DHX15		D				+		
4	DHX36	Probable ATP-dependent RNA helicase DHX36	U					+		
4	DKC1	H/ACA ribonucleoprotein complex subunit B	U	D		D		+		
3	DPP3	Dipeptidyl-peptidase 3		D					+	
3	DYNC1I2	Cytoplasmic dynein 1 intermediate chain 2						+		
3	EBP2	Probable rRNA-processing protein, EBNA1BP2						+		
4	ECH1	Delta(3,5)-Delta(2,4)-dienoyl-CoA isomerase, mitochondrial	U	D	U				+	(33)
2	EEF1A1	Elongation factor 1-alpha 1	U	D	U	D			+	(34)
2	EEF1A2	Elongation factor 1-alpha 2	U		U		Orf3	+		(35)
2	EEF1G	Elongation factor 1-gamma	U	D	U				+	
3	EEF2	Elongation factor 2	U	D	U			+		(36)
16	EFTUD2	116 kDa U5 small nuclear ribonucleoprotein component		D		D		+		(37)
2	EIF2A	Eukaryotic translation initiation factor 2 subunit 1, EIF2							+	
6	EIF3A	Eukaryotic translation initiation factor 3 subunit A	U	D		D		+		(38)
4	EIF3B	Eukaryotic translation initiation factor 3 subunit	U	D		D		+		
3	EIF3CL	Eukaryotic translation initiation factor 3 subunit C-like protein		D		D		+		
2	EIF3E	Eukaryotic translation initiation factor 3 subunit E	U	D	U			+		(39)
7	EIF3L	Eukaryotic translation initiation factor 3, subunit E interacting protein		D				+		
13	EIF4A1	Eukaryotic initiation factor 4A-I, DDX2A	U	D	U	D			+	
2	EIF5A	Eukaryotic translation initiation factor 5A-1	U	D	U	D			+	(40)
2	EMG1	Ribosomal RNA small subunit methyltransferase NEP1	U	D					+	
3	ENO1	Isoform alpha-enolase of Alpha-enolase	U	D	U	D			+	(41)
6	EPHX1	Epoxide hydrolase		D		D		+		(42)
3	ESYT1	Extended synaptotagmin-1						+		(43)
3	EZR	Ezrin	U	D	U	D			+	(44)
3	FKBP4	FK506-binding protein, FKBP52							+	(45)
27	FLNA	Filamin-A	U	D	U	D		+		(46)
25	FLNB	Filamin-B	U		U				+	(29)
9	FLNC	Filamin-C	U	D				+		(47)
2	FTH1	Ferritin heavy chain	U	D	U	D			+	(48)
10	G6PD	Glucose-6-phosphate 1-dehydrogenase (anemia, diabetes)	U	D	U			+		(49)
5	GANAB	Neutral alpha-glucosidase A		D		D			+	(50)
2	GAPDH	Glyceraldehyde-3-phosphate dehydrogenase	U	D		D			+	(51)
2	GAR1	H/ACA ribonucleoprotein complex subunit 1						+		
2	GBE1	1,4-alpha-glucan-branching enzyme	U						+	
4	GCLC	Glutamate-cysteine ligase catalytic subunit (hemolytic anemia)					Orf3		+	
6	GDI1	Rab GDP dissociation inhibitor alpha	U	D	U				+	(52)
7	GDI2	cDNA FLJ60299, highly similar to Rab GDP dissociation inhibitor beta	U	D	U				+	(53)
2	GLO1	Lactoylglutathione lyase (prostasome)		D			Orf3		+	(54)
2	GPC1	Glypican-1		D		D		+		
2	GPI	Glucose-6-phosphate isomerase	U	D	U	D	Orf3		+	(55)
4	GRWD1	Glutamate-rich WD repeat-containing protein 1						+		
6	GSTP1	Glutathione S-transferase	U	D		D			+	(56)
2	H2AFR	Histone H2A type 1-A, H2AC1, HIST1H2AA						+		(57)
3	H2AFV	Histone H2A.V, H2AZ2		D		D		+		(57)
11	H2AFY	H2A histone family, member Y isoform, MacroH2A1	U					+		(58)
2	H2AFY2	Core histone macro-H2A.2, MACROH2A2						+		(57)
4	HADHA	Trifunctional enzyme subunit alpha, mitochondrial						+		
2	HARS	Histidyl-tRNA synthetase, cytoplasmic, HRS							+	(59)
4	HEATR1	HEAT repeat-containing protein 1	U	D	U	D		+		
2	HIST1H1B	Histone H1.5, H1–5	U	D		D		+		(60)
6	HIST1H1C	Histone H1.2	U	D	U	D		+		(60)
2	HIST1H2BL	Histone H2B type 1-L, H2BC13	U	D	U	D		+		(61)
9	HIST1H4J	Histone H4, H4C1	U	D	U	D		+		(62)
12	HIST2H2BE	Histone H2B type 2-E, H2BC21	U	D		D		+		(63)
5	HIST2H3A	HIST2H3C, Histone H3.2, H3C15	U	D	U			+		(64)
3	HNRNPA2B1	Heterogenous nuclear ribonucleoproteins A2/B1	U	D					+	(65)
3	HNRNPC	Heterogeneous nuclear ribonucleoproteins C1/C2	U	D	U	D		+		(66)
6	HNRNPCL1	Heterogeneous nuclear ribonucleoprotein C-like 1						+		
4	HNRNPF	Heterogeneous nuclear ribonucleoprotein F		D		D		+		(67)
2	HNRNPH1	Heterogeneous nuclear ribonucleoprotein H	U	D	U			+		(67)
3	HNRNPK	Heterogeneous nuclear ribonucleoprotein K	U						+	(68)
3	HNRNPM	Heterogeneous nuclear ribonucleoprotein M	U	D		D		+		
4	HNRNPR	Heterogeneous nuclear ribonucleoprotein R	U	D	U				+	(69)
2	HNRNPU	Heterogeneous nuclear ribonucleoprotein U	U	D	U	D		+		
6	HNRNPUL1	hnRNP U-like protein 1	U	D		D		+		
4	HNRNPUL2	hnRNP U-like protein 2	U	D				+		
4	HSP70T	Heat shock 70 kDa protein 1-like, HSPA1L							+	
11	HSP90AA1	Heat shock 90kDa protein 1, alpha isoform	U	D	U				+	(70)
3	HSP90AA2	Heat shock protein HSP 90-alpha A2	U	D	U			+		(71)
5	HSP90AB1	Heat shock protein HSP 90-beta	U	D	U				+	(72)
23	HSP90B1	Endoplasmin	U	D	U			+		(73)
7	HSPA1A	Heat shock 70 kDa protein 1A	U	D	U	D	N, Orf9b		+	(74)
35	HSPA5	HSPA5 protein, BiP, GRP78	U	D	U	D	Nsp2 Nsp4	+		(75)
2	HSP70B	Putative heat shock 70 kDa protein, HSPA7	U	D	U			+		
20	HSPA8	Heat shock cognate 71 kDa protein	U	D	U		Nsp2	+		(76)
17	HSPA9	Stress-70 protein, mitochondrial	U	D		D	N		+	(76)
2	HSPBP1	Hsp70-binding protein	U	D					+	(77)
3	HSPG2	Basement membrane-specific heparan sulfate proteoglycan core protein	U	D	U			+		(78)
4	HTATSF1	HIV Tat-specific factor 1		D		D		+		
4	IDE	Insulin-degrading enzyme (insulin, amyloid)					Nsp4		+	
2	IL18	Interleukin-18	U	D	U	D			+	(79)
5	ILF2	Interleukin enhancer-binding factor	U		U			+		(80)
9	IQGAP1	Ras GTPase-activating-like protein IQGAP1	U					+		(81)
4	ITGB1	Integrin beta-1	U	D		D	Nsp4 Orf8	+		(82)
2	IWS1	Protein IWS1 homolog	U	D	U	D		+		
3	KARS	Lysyl-tRNA synthetase						+		(83)
10	KPNB1	Importin subunit beta-1, IPOB,						+		(84)
2	KRR1	KRR1 small subunit processome component homolog, HIV-1 Revbinding protein						+		(85)
2	KYNU	Kynureninase	U		U		Orf3		+	
2	LAMP2	Lysosome-associated membrane glycoprotein 2	U	D	U	D			+	(86)
2	LARS	Leucyl-tRNA synthetase, cytoplasmic						+		(83)
3	LCP1	Plastin-2	U	D					+	(87)
8	LDHA	L-lactate dehydrogenase	U	D	U	D			+	(88)
9	LDHB	L-lactate dehydrogenase B chain	U	D	U	D			+	(88)
2	LEO1	RNA polymerase-associated protein LEO1	U		U			+		
6	LMNA	Lamin-A/C	U	D	U	D			+	(89)
2	LMNB2	Lamin-B	U	D				+		(90)
4	LRPPRC	Leucine-rich PPR motif-containing protein, mitochondrial		D					+	(91)
3	MAP1B	Microtubule-associated protein 1B	U	D	U	D		+		(92)
2	MARS	Methionyl-tRNA synthetase, cytoplasmic, SLA2		D		D		+		
3	MDH2	Malate dehydrogenase, mitochondrial	U	D					+	(93)
4	MOV10	Helicase MOV-10, Moloney Leukemia virus 10 protein	U	D		D	N	+		
5	MRPL1	39S ribosomal protein L1, mitochondrial						+		
3	MRPL13	39S ribosomal protein L13, mitochondrial		D		D		+		
2	MRPL15	39S ribosomal protein L15, mitochondrial	U	D		D		+		
2	MRPL17	39S ribosomal protein L17, mitochondrial		D		D		+		
2	MRPL18	39S ribosomal protein L18, mitochondrial		D		D		+		
4	MRPL19	39S ribosomal protein L19, mitochondrial		D		D		+		
2	MRPL2	39S ribosomal protein L2, mitochondrial		D		D		+		
2	MRPL23	39S ribosomal protein L23, mitochondrial		D				+		
5	MRPL37	39S ribosomal protein L37, mitochondrial	U	D		D		+		
5	MRPL38	39S ribosomal protein L38, mitochondrial		D		D		+		
2	MRPL39	39S ribosomal protein L39, mitochondrial		D		D		+		
3	MRPL45	39S ribosomal protein L45, mitochondrial		D		D		+		
2	MRPL49	39S ribosomal protein L49, mitochondrial		D		D		+		
4	MRPS22	28S ribosomal protein S22, mitochondrial						+		
4	MRPS23	28S ribosomal protein S23, mitochondrial						+		
6	MRPS27	28S ribosomal protein S27, mitochondrial					Nsp8	+		
2	MRPS28	28S ribosomal protein S28, mitochondrial, MRPS35						+		
6	MRPS29	28S ribosomal protein S29, mitochondrial, DAP3						+		
2	MRPS30	28S ribosomal protein S30, mitochondrial		D		D		+		
2	MRPS34	28S ribosomal protein S34, mitochondrial		D				+		
16	MRPS39	Pentatricopeptide repeat-containing protein 3, mitochondrial, PTCD3						+		
3	MRPS9	28S ribosomal protein S9, mitochondrial						+		
4	MSN	Moesin (membrane organizing extension spike protein)	U		U		Nsp6O rf3		+	(94)
12	MVP	Major vault protein	U	D	U			+		(95)
16	MYBBP1A	Myb-binding protein 1A	U	D	U			+		
2	MYG1	UPF0160 protein MYG1, mitochondrial							+	
14	MYH9	Myosin-9	U	D	U	D			+	(96)
2	MYO1E	Unconventional myosin-Ie, MYO1C	U					+		(97)
3	NAP1L1	Nucleosome assembly protein 1-like 1	U	D		D		+		
18	NCL	Nucleolin	U	D				+		(98)
8	NME1	Nucleoside diphosphate kinase	U	D		D			+	(99)
3	NME2	Nucleoside diphosphate kinase 2, NM23	U	D					+	(100)
2	NOC2L	Nucleolar complex protein 2 homolog		D		D		+		
9	NOP2	Probable 28S rRNA (cytosine(4447)-C(5)-methyltransferase	U		U			+		
2	NPEPPS	Puromycin-sensitive aminopeptidase, metalloproteinase MP100							+	
6	NPM1	Nucleophosmin	U	D	U	D		+		(101)
2	NRCAM	Neuronal cell adhesion molecule	U	D	U	D			+	(102)
4	NUDT21	Cleavage and polyadenylation specificity factor subunit 5		D		D			+	
2	OLA1	Obg-like ATPase 1	U		U				+	
11	P4HB	Protein disulfide-isomerase	U	D	U	D			+	(103)
22	PABPC1	Polyadenylate-binding protein 1		D		D	N	+		(104)
11	PABPC4	Polyadenylate-binding protein 4		D		D	N	+		(105)
4	PAF1	RNA polymerase II-associated factor 1 homolog		D				+		
2	PAFAH1B2	Platelet-activating factor acetylhydrolase IB subunit beta	U	D	U	D			+	
3	PAFAH1B3	Platelet-activating factor acetylhydrolase IB subunit gamma	U		U				+	
6	PCNA	Proliferating cell nuclear antigen	U	D	U	D			+	(106)
12	PDIA3	Protein disulfide-isomerase A3	U	D	U		Orf8		+	(107)
18	PDIA4	Protein disulfide-isomerase A4	U	D	U				+	
10	PDIA6	Protein disulfide-isomerase A6	U	D	U	D			+	(108)
6	PELP1	Proline-, glutamic acid-, leucine-rich protein 1		D		D		+		
2	PES1	Pescadillo homolog		D				+		
2	PFAS	Phosphoribosylformylglycinamidine synthase, GATD8, FGARAT, PURL							+	
2	PFKP	ATP-dependent 6-phofructokinase, platelet type	U	D	U		Orf7a		+	(109)
2	PGAM2	Phosphoglycerate mutase, PGAMM							+	
9	PGD	6-phosphogluconate dehydrogenase, decarboxylating	U	D	U				+	
2	PLD3	Phospholipase D3, 5’−3’ exonuclease PLD3	U	D		D	Nsp2O rf8 Orf7b		+	
39	PLEC	Plectin-1	U	D	U	D		+		(110)
2	PLS1	Plastin-1		D					+	
5	PLS3	Plastin-3	U	D					+	
2	POP1	Ribonucleases P/MRP protein subunit POP1	U					+		(111)
3	POR	NADPH--cytochrome P450 reductase	U	D	U		Nsp2	+		
2	PPA1	Inorganic pyrophosphatase (Guillain-Barre syndrome)	U				Orf3		+	(112)
5	PPIB	Peptidyl-prolyl cis-trans isomerase	U	D	U				+	(113)
6	PRDX1	Peroxiredoxin-1	U	D	U	D			+	(114)
4	PRDX3	Thioredoxin-dependent peroxide reductase, mitochondrial	U	D	U				+	(115)
3	PRDX4	Peroxiredoxin-4	U	D					+	(116)
17	PRKDC	DNA-dependent protein kinase catalytic subunit	U	D	U		M Nsp4	+		(117)
3	PRMT1	HMT1, hnRNP methyltransferase-like 2 isoform		D		D			+	
19	PRPF8	Pre-mRNA-processing-splicing factor 8	U	D	U	D		+		(29)
6	PSAT1	Phosphoserine aminotransferase 1	U	D	U		Orf3 Orf7a		+	
2	PSMD1	26S proteasome non-ATPase regulatory subunit 1	U		U			+		
2	PSMD6	26S proteasome non-ATPase regulatory subunit 6, PFAAP4							+	
18	PUM1	Pumilio homolog 1		D		D		+		
3	PURA	Transcriptional activator protein Pur-alpha	U	D	U	D		+		
2	PURH	Bifunctional purine biosynthesis protein, ATIC							+	(118)
4	QARS	Glutaminyl-tRNA synthetase						+		(83)
3	RAB1A	Ras-related protein Rab-1A (intracellular membrane trafficking)		D			Nsp7 Orf3 Orf7b	+		
6	RALY	RNA-binding protein, autoantigen p542	U	D	U	D		+		(119)
2	RANGAP1	Ran GTPase-activating protein 1		D				+		(120)
3	RARS	Arginyl-tRNA synthetase, cytoplasmic, RARS1	U		U			+		
5	RBBP4	Histone-binding protein RBBP4		D				+		(121)
3	RBM8A	RNA-binding protein 8A	U					+		
8	RDX	Radixin					Nsp13 Orf3		+	(122)
3	RNPEP	Arginine aminopeptidase, APB							+	
2	RNPS1	RNA-binding protein with serine-rich domain 1	U	D	U	D		+		
4	RO60	60 kDa SS-A/Ro ribonucleoprotein	U		U			+		(123)
3	RPF2	Ribosome production factor 2 homolog, BXDC1						+		
4	RPL10A	60S ribosomal protein L10A, NEDD6						+		
2	RPL11	60S ribosomal protein L11	U		U			+		
4	RPL12	60S ribosomal protein L12	U	D	U			+		(124)
3	RPL15	60S ribosomal protein L15		D		D		+		
3	RPL18	60S ribosomal protein L18		D		D		+		
2	RPL26L1	60S ribosomal protein L26-like 1, RPL26P1						+		
2	RPL35A	60S ribosomal protein L35a	U	D	U			+		(125)
2	RPL4	60S ribosomal protein L4	U	D	U	D		+		
17	RPL5	60S ribosomal protein L5		D		D		+		(126)
11	RPL6	60S ribosomal protein L6	U	D	U	D		+		(127)
9	RPL7	60S ribosomal protein L7, RPL7P32	U	D	U	D		+		(128)
4	RPL7A	60S ribosomal protein L7A	U	D		D		+		(125)
2	RPL8	60S ribosomal protein L8	U	D	U	D		+		(120)
8	RPLP0	60S acidic ribosomal protein P0	U	D	U			+		(129)
2	RPLP1	60S acidic ribosomal protein P1	U	D		D		+		(130)
3	RPLP2	60S acidic ribosomal protein P2	U	D	U	D		+		(130)
2	RPS15A	40S ribosomal protein S15a	U					+		
3	RPS18	40S ribosomal protein S18	U	D				+		(120)
3	RPS2	40S ribosomal protein S2	U	D	U	D		+		
3	RPS3	40S ribosomal protein S3	U	D	U			+		(131)
4	RPS4X	40S ribosomal protein S4, X isoform		D		D		+		
3	RPS6	40S ribosomal protein S6	U	D	U	D		+		(125)
2	RPS8	40S ribosomal protein S8	U	D	U			+		
8	RPS9	40S ribosomal protein S9		D				+		(125)
4	RPSA	40S ribosomal protein SA, LMAR1							+	(132)
2	RRBP1	Ribosome-binding protein 1	U	D		D		+		
11	RRP12	RRP12-like protein	U					+		
3	RRP9	U3 small nucleolar RNA-interacting protein 2 (U3–55K)	U	D			N	+		(133)
4	RRS1	Ribosome biogenesis regulatory protein homolog	U		U			+		
5	RSL1D1	Ribosomal L1 domain-containing protein 1	U	D	U			+		
2	RUVBL1	RuvB-like 1						+		(134)
13	SAP130	Histone deacetylase complex subunit SAP130	U		U			+		
5	SERPINB1	Leukocyte elastase inhibitor	U						+	
4	SERPINB6	Serpin B6, peptidase inhibitor 6							+	
2	SERPINC1	Antithrombin-III	U						+	
6	SET	Protein SET, phosphatase 2A inhibitor	U	D		D		+		
14	SF3B1	Splicing factor 3B subunit 1	U	D		D		+		(135)
8	SFN	14-3-3 protein sigma	U	D	U				+	(136)
2	SLC1A5	Neutral amino acid transporter B, Simian type D retrovirus receptor, Baboon M7 virus receptor	U	D	U			+		
2	SLC2A1	HepG2 glucose transporter, human T-cell leukemia virus receptor, GLUT1		D		D	Nsp8	+		(137)
17	SLC3A2	4F2 cell-surface antigen heavy chain	U	D	U			+		
2	SND1	Staphylococcal nuclease domain-containing protein 1	U	D		D			+	
15	SNRNP200	U5 small nuclear ribonucleoprotein 200 kDa helicase		D		D		+		(138)
3	SNRPA	U1 small nuclear ribonucleoprotein A	U					+		(139)
2	SNRPB	Small nuclear ribonucleoprotein-associated proteins B and B’	U	D	U	D		+		(140)
2	SNRPD1	Small nuclear ribonucleoprotein Sm D1	U		U			+		(141)
4	SNRPD2	Small nuclear ribonucleoprotein Sm D2		D		D		+		(142)
2	SNRPG	Small nuclear ribonucleoprotein G, PBSCG						+		(Satoh, Chan et al.
2	SOD1	Superoxide dismutase [Cu-Zn]	U	D					+	(143)
46	SPTAN1	Spectrin alpha chain, brain	U	D		D		+		(144)
29	SPTBN1	Spectrin beta chain, brain 1	U	D	U	D		+		(145)
2	SRP72	Signal recognition particle 72 kDa protein		D		D		+		(146)
3	SRSF1	Serine arginine rich splicing factor 1, ASF, SF2	U	D		D		+		(147)
6	SSB	Lupus La protein	U	D	U	D			+	(59)
9	SSBP1	Single-stranded DNA-binding protein, mitochondrial					N	+		
8	SSRP1	FACT complex subunit SSRP1	U	D	U	D		+		(148)
2	ST13	Hsc70-interacting protein	U		U				+	(149)
9	SUPT16H	FACT complex subunit SPT16		D		D		+		
2	SUPT5H	Transcription elongation factor SPT5						+		
2	SYNCRIP	Heterogeneous nuclear ribonucleoprotein Q		D		D		+		
11	TALDO1	Transaldolase	U	D	U	D			+	(150)
4	TEX10	Testis-expressed protein 10						+		
3	TFG	TRK-fused gene protein							+	
4	TGM2	Protein-glutamine gamma-glutamyltransferase 2	U	D	U	D			+	(151)
4	TLN1	Talin-1	U	D	U				+	(152)
3	TOP1	DNA topoisomerase 1	U					+		(153)
5	TP53I3	Quinone oxidoreductase	U	D	U	D			+	
7	TPM1	Tropomyosin 1 alpha chain	U	D	U	D			+	(154)
2	TPM2	Tropomyosin beta chain	U	D	U	D			+	
4	TPM3	Tropomyosin alpha-3 chain	U	D	U	D			+	(155)
8	TPM4	Tropomyosin alpha-4 chain	U	D	U				+	(156)
3	TSN	Translin		D		D			+	
4	TUBA1C	Tubulin alpha-1C chain	U	D	U	D		+		(157)
5	TUBA4A	Tubulin alpha-4A chain	U	D		D		+		(158)
4	TUBB4B	Tubulin beta-2C chain, TUBB2C	U	D	U			+		(159)
2	TXNDC5	Thioredoxin domain-containing protein 5	U	D	U	D			+	
15	TXNRD1	Thioredoxin reductase 1, cytoplasmic	U	D	U				+	(160)
7	UBA1	Ubiquitin-like modifier-activating enzyme 1	U	D	U	D			+	(161)
3	UBC	RPS27A; UBB ubiquitin-40S ribosomal protein S27a precursor	U	D				+		(162)
2	UBTF	Nucleolar transcription factor 1, autoantigen NOR-90		D		D		+		(163)
2	UCHL1	Ubiquitin carboxyl-terminal hydrolase isozyme L1	U	D	U	D	Orf3		+	(164)
5	UGDH	UDP-glucose 6-dehydrogenase	U	D	U				+	
15	UPF1	Regulator of nonsense transcripts 1		D			N	+		
2	USP7	Ubiquitin carboxyl-terminal hydrolase (Herpes virus associated)	U		U			+		
27	VCL	Vinculin	U	D	U				+	(165)
18	VCP	Transitional endoplasmic reticulum ATPase	U	D	U	D			+	(166)
11	VIM	Vimentin	U	D	U	D		+		(167)
5	WDR18	WD repeat-containing protein 18		D				+		
32	XRCC5	ATP-dependent DNA helicase 2 subunit 2		D		D		+		(168)
30	XRCC6	ATP-dependent DNA helicase 2 subunit 1	U	D	U	D		+		(169)
3	YBX1	Y-box-binding protein 1	U	D	U	D		+		(170)
6	YBX3	Y-box binding protein 3, CSDA	U	D	U	D		+		(171)
5	YWHAB	14-3-3 protein beta/alpha	U	D		D			+	
15	YWHAE	14-3-3 protein epsilon	U	D	U	D			+	(136)
6	YWHAG	14-3-3 protein gamma	U	D					+	(136)
3	YWHAH	14-3-3 protein eta		D					+	(172)
7	YWHAQ	14-3-3 protein theta	U	D	U				+	(132)
7	YWHAZ	14-3-3 protein zeta/delta	U	D		D			+	(173)

Notes:

# Pep.: number of peptides identified by mass spectrometry; COVID (up/down): protein or gene expression up- and/or down-regulated in SARS-Cov-2 infected cells or patients; A549 infection (up/down): protein or gene expression up- and/or down-regulated in SARS-Cov-2 infected A549 cells; Interactome: host protein interacting with SARS-CoV-2 protein; DS-affinity: concentration of NaCl (1.0 M, strong affinity, or 0.5 M, intermediate affinity) at which a DS-binding protein elutes from DS-affinity resin.
